# First-in-human experience from RESET-AF *(R*apid on*E*-*S*hot *E*lectroporation *T*rial for *A*trial *F*ibrillation)

**DOI:** 10.1007/s10840-026-02434-3

**Published:** 2026-09-17

**Authors:** Paul A. Gould, Stuart P. Thomas, Waheed Ahmad, Pierre C. Qian, Arash Aryana

**Affiliations:** 1 Princess Alexandra Hospital, Brisbane, QLD Australia; 2 Westmead Hospital, Westmead, NSW Australia; 3 Mercy General Hospital and Dignity Health Heart and Vascular Institute, Sacramento, CA USA

**Keywords:** Atrial fibrillation, Catheter ablation, ElePulse, Pulmonary vein isolation, Pulsed field ablation

## Abstract

**Background and Aims:**

Pulsed field ablation (PFA) is increasingly used to treat patients with atrial fibrillation (AF). The RESET-AF was a single-arm, first-in-human, clinical trial of AF ablation using a novel, single-shot PFA system (ElePulse^®^, CRC EP, Tustin, CA, USA), trial registration ACTRN12622001523796 (Date registered 8th December 2022).

**Methods:**

In 36 consecutively-enrolled patients (57 ± 11 years, 69% male) with symptomatic paroxysmal (*n* = 22) and persistent (*n* = 14) AF, pulmonary vein isolation (PVI) was performed using this catheter, guided by 3D mapping. All patients underwent remapping at 3 months to confirm PVI durability. Patients were followed at 6 and 12 months with clinical review, Holter monitoring and electrocardiography. The primary efficacy and safety endpoints included a freedom from recurrent AF at 12 months > 50% and serious adverse events (SAE) at 7 and 30 days < 5%, respectively. The secondary efficacy endpoints included 3-month PVI durability > 50% and 6-month freedom from AF > 60%.

**Results:**

All endpoints were achieved in the entire study cohort. There was 1 SAE (renal impairment which resolved with conservative management) with a total SAE rate of 2.8%. One patient was lost to follow-up at 12 months with a primary efficacy endpoint of 92% (32/35 patients). The secondary efficacy endpoints were 82% 3-month PVI and 94% (34/36 patients) 6-month freedom from AF. During the study, the PFA dose was adjusted in 3 cohorts. The “optimized subset” had 100% PVI durability and freedom from AF at 6 and 12 months without SAEs.

**Conclusion:**

This study of a novel PFA catheter system for ablation of paroxysmal and persistent AF achieved all predetermined primary and secondary clinical endpoints.

**Supplementary Information:**

The online version contains supplementary material available at https://doi.org/10.1007/s10840-026-02434-3.

## What’s New

This study examined a novel spiral “single shot” PFA ablation catheter which used a standard 8.5 Fr guidance sheath to access the left atrium and perform pulmonary vein isolation in a FIH study of 36 patients (22 with paroxysmal AF and 14 with persistent AF)

The novel spiral PFA catheter employed in this study is not over the wire and once positioned in a vein does not require repositioning to achieve isolation

This study was one of the earliest using real-time impedance during PFA delivery to monitor electrode-tissue contact.

This study used a PFA generator capable of detecting electrode collision, thereby preventing arcing. The operator had the ability to deselect quickly the colliding electrodes and move on with patient treatment.

As it was conducted in 2023, this study was likely the first human PFA study to present data from completely zero-fluoro cases and one the earliest PFA studies to present remap data at 3 months for all patients enrolled.

**Supplementary Information:**

The online version contains supplementary material available at https://doi.org/10.1007/s10840-026-02434-3.

## Introduction

Pulse field ablation (PFA) is an emerging therapeutic strategy for the treatment of paroxysmal (PAF) [1] and persistent (PersAF) [2, 3] atrial fibrillation (AF) with growing evidence in support of its efficacy as compared to conventional thermal modalities [4]. The most studied are the Farapulse (Boston Scientific, Marlborough, MA) [4] and PulseSelect (Medtronic, Minneapolis, Minnesota) [5]. Each system differs in its characteristics of PFA delivery. However, these available PFA systems are similar in that they require an over the wire technique and multiple manoeuvres within each pulmonary vein (PV) to cover the antral portion to achieve acute PV isolation (PVI). The ablation catheter movement within each PV is not precise and raises the possibility of reversible electroporation lesions which will decrease PVI durability and hence long-term success.

**Fig. 1:**
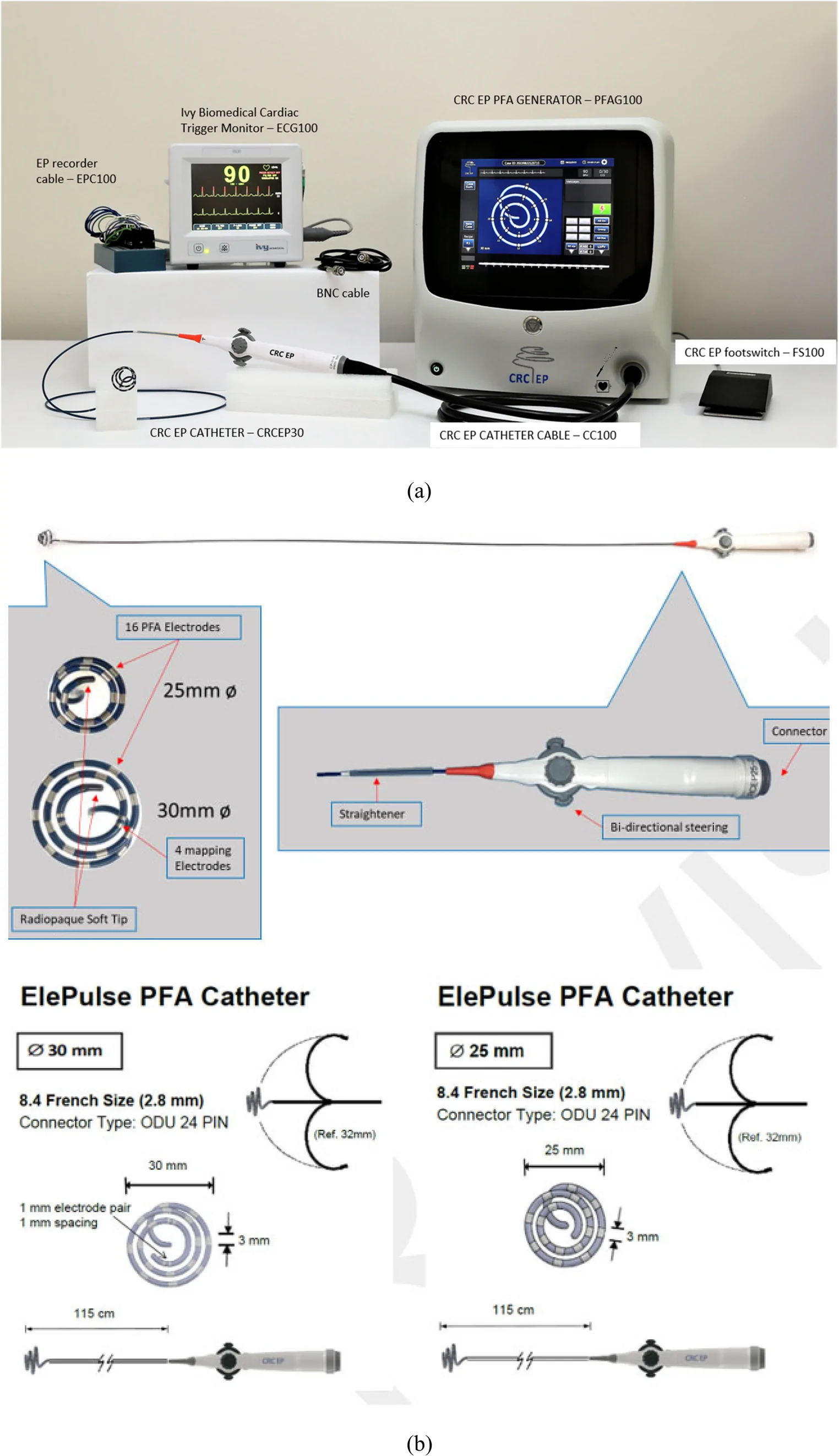
ElePulse PFA System. Image (**a**) shows the ElePulse PFA system (CRC EP, California, USA) components, including the ElePulse PFA catheter, trigger monitor, and ElePulse PFA generator and accessories. Image (**b**) details the 25-mm and 30-mm ElePulse PFA catheters. Alt text: (**a**) Image of ElePulse PFA system with the following components: ElePulse PFA Generator PFAG100, ElePulse PFA Catheter CRCEP30, ElePulse PFA Catheter Cable CC100, CRC EP footswitch FS100, Ivy Biomedical Cardiac Trigger Monitor ECG100, EP recorder cable EPC100, and BNC cable. (**b**) Figure detailing the main components of both the 25-mm and 30-mm sizes for the ElePulse PFA catheters

Accordingly, in the Rapid onE-Shot Electroporation Trial for Atrial Fibrillation (RESET-AF), we present the first-in-human (FIH) experience using a novel PFA catheter and system (ElePulse^®^, CRC EP, Tustin, CA) for PVI. The ElePulse PFA Catheter differs from other available PFA catheters due to its single-shot, not-over-the-wire design for single-position treatment delivery. Durability of PVI was assessed using re-mapping studies at 3 months and clinical follow-up at 6 and 12 months as adopted in other first-in-human studies [5]. We have previously published preclinical data from acute and chronic animal studies using the same PFA catheter system which has demonstrated durable transmural lesions in the porcine atria and ventricles out to 3 months [6, 7]. From these data, we developed our parameters for the human PFA treatment to achieve irreversible electroporation and durable PVI. The primary objective of the RESET-AF study was to assess the safety and effectiveness of the ElePulse system (Fig. 1) in humans for the treatment of drug refractory, symptomatic PAF and persAF.

## Methods

The RESET-AF study was a prospective, multicentre, FIH study. The trial enrolled 36 subjects (PAF = 22 and PersAF = 14) consecutively, at 2 clinical sites in Australia: the Princess Alexandra Hospital, Brisbane, and the Westmead, Hospital, Sydney.

The study endpoints are presented in Supplemental Table (1) These endpoints were similar to those used for other technologies in their proof-of-concept FIH studies [1]. Serious adverse events (SAE) were those established by each of the regulatory committees of North America, Europe and Southeast Asia [8]. The study inclusion and exclusion criteria are presented in Supplemental Table (2) The data collected for each subject undergoing a procedure are presented in Supplemental Table 3.

### ElePulse PFA system

PFA was performed using bipolar, non-irrigated, ElePulse PFA catheters and a custom programmable PFA generator (Fig. 1a and b). The catheters were 8.4-French shaft, 16-electrode, bidirectional, 25- or 30-mm diameter designs with radiopaque tips, intended for single-shot ablation. There is no need for the repositioning of the ElePulse catheter between treatments within PVs. Additionally, mapping and contact parameters (impedance-based) were used to guide ablation. When positioned at the PV antra, the mapping electrodes allow the operator to record the PV potentials during PFA to provide real-time assessment of PV isolation (i.e., “entrance block”). Alternatively, pacing was performed using these electrodes to evaluate for “exit block.” The position of these electrodes on the electroanatomic map (EAM) during delivery of PFA energy confirmed catheter stability. Trans-esophageal or intracardiac echo modalities were also used to confirm catheter placement. Moreover, three-dimensional EAM can be performed using the ElePulse PFA catheter, itself, when using impedance-based mapping systems (EnSite, Abbott, Chicago, IL or Carto, J&J MedTech, Irvine, CA). During PFA, energy was typically delivered simultaneously via all 16 electrodes unless one or more were noted to be colliding. In that case, the generator allowed for PFA through individually selected electrodes. Figure 1 illustrates the design of the two PFA/mapping catheters and systems. The left atrium was accessed through commercially available, 8.5-French steerable introducer. The system delivered bipolar, biphasic, microsecond-wide pulses with amplitudes > 2 kV. Although energy delivery was spread over 30 s to minimize thermal effects, the waveform is designed such that the actual “PFA-on” time is < 2 ms. The generator automatically terminated energy delivery upon detecting electrode collision, guided by real-time impedance checks to circumvent arcing. In such a situation, the generator provided the option of isolating the colliding electrodes into separate groups sequentially energized. Lastly, pulsed field applications gated with the QRS using a cardiac trigger monitor (Model 7600, Ivy Biomedical Systems, Branford, CT) avoiding delivery during the vulnerable period.

### Procedural details

Prior to the procedure, patients underwent a cardiac computed tomography scan with contrast using the standard protocol for each institution and trans-esophageal or intra-cardiac echocardiography was performed on the day/day prior to the procedure a to exclude left atrial thrombus. On the day of the procedure, all patients underwent general anesthesia without paralysis and the right femoral vein (RFV) was accessed using an 11-Fr sheath (Abbott) or directly using a commercially-available, 8.5-Fr, steerable introducer (Agilis, Abbott). In addition, a coronary sinus catheter was sited via a 7-Fr RFV sheath.

A single transseptal puncture was performed (Brockenbrough needle, Medtronic, Minnesota). Heparin was administered intravenously, titrated to an activated clotting time (ACT) of 350–450 s and an esophageal temperature probe was inserted. A circular mapping catheter (Abbott) was subsequently passed into the left atrium to create a baseline, high-density (> 4000 points) left atrial EAM (Ensite X, Abbott).

Subsequently, the ElePulse PFA catheter was positioned at the PV antra. Once a suitable position was achieved, tissue contact was assessed by impedance (> 150 Ω) and pacing capture at 10 mA at 2 msec on all 16 electrodes. Once the catheter position and contact were satisfactory, 1 to 2 PFA applications were delivered to each PV with an amplitude > 2 kV for 30–60 cardiac cycles. The ElePulse catheter was positioned at a single site/PV antra and only repositioned to target another PV. The anatomical locations of each PFA application were shadowed on the EAM. Post-ablation, a repeat, dense EAM was recreated following a 20-min waiting period (> 4000 points) using the circular mapping catheter, while confirming entrance and exit block using pacing maneuvers in all PVs. If conduction resumed in any of the PVs, additional PFA applications were delivered until PVI was achieved. PFA was delivered by the PFA generator according to alternate pulse train configurations confirmed in preclinical studies [6, 7]. There was no direct skeletal muscle capture observed. When ablating around the RSPV, phrenic nerve mediated chest movements were occasionally noted. There was minimal impact to EAMs.

### Contact parameters and PV electrograms

Close spacing of the 16 electrodes (8 pairs) on the PFA catheter, enabled clear nearfield electrograms measurement with the ability to nearfield pace in each PV. Prior to each PFA application, contact was assessed by way of pacing capture (10 mA at 2.0 msec) from all 16 PFA electrodes (8 pairs) along with local impedance > 150 Ω on all 16 electrodes.

### Treatment dosage cohorts and intravenous hydration

There were 3 patient cohorts assessed in this study based on PFA dosage and hydration. This was designed to determine the optimal dosage to achieve PVI at 3 months and freedom from recurrent AF, and to avoid hemolysis. The first and second cohorts received “high” (between 2 and 3 kV) and “low-dose” (2 kV) PFA, respectively. The first cohort had no hydration and the second cohort was hydrated as per the operator’s discretion. The first cohort received treatments for 60 cycles on two occasions without catheter repositioning between applications. The second cohort received treatments for 60 cycles for a single treatment in each PV.

The third cohort was conceived after reviewing the results of the second cohort with respect to decreased durability. It received “medium-dose” PFA (between 2 and 3 KV) with treatments for 30 cardiac cycles and two treatments/PV without catheter repositioning in between applications with rigorous intravenous hydration protocol (a 200 mL bolus of intravenous normal saline on anesthetic induction, followed by 200 mL/h during and 1000 mL of normal saline post-procedure during the ensuing 24 h). The basis for the cohort doses and hydration protocol came from unpublished animal data please see Supplemental Fig. 1.

### Post-procedure follow-up

Post-procedure, patients were clinically reviewed immediately on day 1 and at 3, 6, and 12 months. At each clinical review, an electrocardiogram was performed along with 24-hour Holter monitors at 6 and 12 months. Laboratory tests investigating for hemolysis and renal function including a complete blood count, creatinine, urea, glomerular filtration rate [GFR], electrolytes, LDH, haptoglobin and liver function were analyzed at baseline prior to the procedure, on day 1, and on day 5–7 post-procedure in cohorts 2 and 3.

### 3-month repeat mapping studies

A follow-up re-mapping study was performed at 3 months under general anesthesia or deep sedation. Briefly, single transseptal catheterization was performed and a circular mapping catheter (Abbott) was used to create a dense EAM (> 4000 points) of the left atrium (Ensite X, Abbott). Pacing maneuvers to confirm entrance and exit block were also performed to assess PVI for each PV. Further ablation to isolate reconnected PVs could be performed using radiofrequency ablation.

### Statistical analysis

Patients were included in either Cohort 1, Cohort 2, or Cohort 3 sequentially as they enrolled in the study (as explained in the Results section). To improve safety and lesion durability, the PFA energy and use of hydration were adjusted over the study enrolment, ending with the most optimized treatment for Cohort 3. The study cohorts included the total cohort pooled data and the Cohort 3 data. Being a first-in-human study, there was no formal hypothesis testing or power calculation. *Post hoc* analyses compared Cohort 3 vs. Cohort 1 and Cohort 2 data to elucidate the differences observed with use of hydration, energy evolution and optimization of the PFA treatment.

Continuous variables are summarized as mean ± SD and comparisons among cohorts were provided. Categorical variables are summarized as number and percentage. Analyses were conducted using SAS version 9.4 (SAS Institute). The study was intended to be proof-of-concept. Its population size was insufficient to perform statistical significance analyses.

## Results

**Fig. 2:**
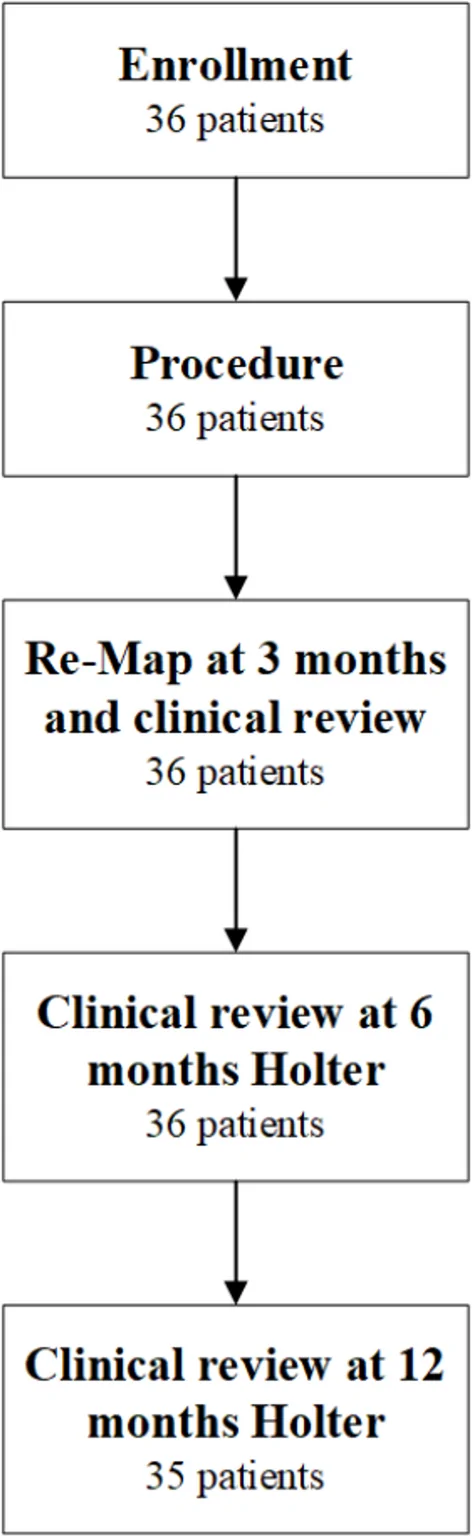
Consort Flow Diagram of Study. The figure outlines subject participation at each stage of the study. Alt text: Diagram outlining subject participation at the following sequential stages: Enrollment, procedure, re-map at 3 months and clinical review, Clinical review at 6 months Holter, and Clinical review at 12 months Holter. 36 patients participated in each stage until the clinical review at 12 months when 35 patients participated

The baseline characteristics of the 36 patients enrolled are presented in Table 1. A consort diagram detailing subject participation is depicted in Fig. 2. Altogether, 36 patients were enrolled with 35 completing 12 months of follow-up (1 patient was lost to follow-up at 12 months). All 36 patients underwent repeat mapping studies at 3 months post-index procedure and 6 months of follow-up.

**Table 1:** Baseline Patient Characteristics^a^

Patient Characteristics	Average/Absolute	%
Demographics		
Age (Years)	56.9 ± 10.7	
Male	25	69
Heart Characteristics		
Left Ventricular Ejection Fraction (%)	56.7 ± 5.9	
Left Atrial Diameter (cm)	4.2 ± 0.5	
Medical History		
Hypertension	14	39
Diabetes	2	6
Stroke or TIA	0	0
GFR (ml/min/1.73m^2^)	89.3 ± 20.0	
Anticoagulation		
Warfarin	0	0
NOAC	36	100
Aspirin	3	8
Antiarrhythmics		
Class I	14	39
Class II	17	47
Class III	16	44
Class V	8	22
None	2	6

^a^*GFR* glomerular filtration rate, *NOAC* novel oral anticoagulant, *TIA* transient ischemic attack

### Procedural characteristics and dosage cohorts

The outcome data for the overall cohort, dosages and the optimal cohorts are presented in Table 2 with additional procedural details in Table 3. All patients achieved acute isolation in all four PVs. In the overall cohort of 36 patients, AF recurrence was encountered in 2 patient (94% freedom from AF) on Holter monitoring at 6 months and AF recurrence in 3 patients (92% freedom from AF) on Holter monitoring at 12 months of follow-up. Overall, PVI durability was present in 82% of the PVs and all PVs remained isolated in 67% of the patients. AF recurrence was defined as the detection of atrial fibrillation episodes of any duration on 12-lead ECG or on 24-hour Holter Monitoring. The efficacy rates above reflect the index procedure and re-ablation of several patients at the treating physician’s discretion when the 3-month remapping was performed.

**Table 2:** Procedure Outcomes for Overall, High Medium and Low Dosage and Optimal Cohorts^a^

Item	Cohort 1 (High Dose)	Cohort 2 (Low Dose)	Cohort 3 (Medium Dose)	Overall (Dose per cohort)	Optimal subset^b^ (Medium Dose)
Number of patients	18	10	8	36	Last consecutive 6
Hydration	No	No	Rigorous	As per cohort	Rigorous
Acute PVI	100%	100%	100%	100%	100%
Serious Adverse Events	1	0	0	1	0
Safety at 7 & 30 days	Yes	Yes	Yes	Yes	Yes
PVI durability at 3 months	92%	55%	94%	82%	100%
Patients with durable PVI at 3 months	78%	40%	75%	67%	100%
Freedom from AF at 6 months (*N* = 36)	100%	80%	100%	94%	100%
Freedom from AF at 12 months (*N* = 35)	94%	89%	100%	92%	100%

^a^*AF* atrial fibrillation, *PVI* pulmonary vein isolation

^b^ Treatment protocol derived from cohort 3 with improved guidance for catheter placement

**Table 3:** Procedure Times for Overall and Dosage Cohorts^a^

Item	Cohort 1	Cohort 2	Cohort 3	Overall study
Duration				
EP procedure time (min)	123 ± 37	143 ± 29	125 ± 43	128 ± 36
Catheter dwell time (min)	58 ± 32	72 ± 34	62 ± 23	63 ± 31
Mapping time (min)	37 ± 16	36 ± 9	25 ± 7	34 ± 13
Total ablation time (min)	6.8 ± 1.4	5.6 ± 2.4	5.3 ± 1.4	6.1 ± 1.8
Radiation				
Total fluoroscopy exposure	20 ± 19	32 ± 19	19 ± 23	23 ± 20
Ablation				
Number of PFA shots/PV	2.1 ± 0.4	1.7 ± 0.7	2.6 ± 0.6	2.1 ± 0.6

Data includes procedure time for overall, left atrial dwell, ablation, mapping, and x-ray

^a^*EP* electrophysiology, *PFA* pulsed-field ablation, *PV* pulmonary vein

Cohort 1 included 18 consecutively-enrolled patients who were ablated with “high dose” PFA (between 2 and 3 KV) without hydration for 60 cardiac cycles on two occasions (same catheter position with each ablation, no intervening catheter repositioning) per each treatment. This approach yielded 92% PVI durability at 3 months with 78% of the patients having durable isolation of all PVs. There was one major SAE in this cohort (refer to Primary Safety Endpoint below). Consequently, it was decided to switch to lower dose treatment regimens with periprocedural hydration, and to alter the inclusion criteria with respect to the GFR (exclusion criteria from GFR < 30 mL/min to < 60 mL/min and no contrast load on the day of the procedure).

Cohort 2 consisted of “low-dose” PFA (~ 2 KV) and included the next 10 consecutive patients treated with a single 60-cardiac cycle application for a single treatment only. PVI durability was 55% with 40% of the patients having all pulmonary veins isolated at 3 months, whereas freedom from recurrent AF were 89% and 80% at 6 and 12 months, respectively without any SAEs.

Cohort 3 had PVI durability of 94% with 75% of patients having all PVs isolated at 3 months. There was a 100% freedom from AF at 6 and 12 months, respectively, and no SAEs. Meanwhile, detailed review of EAMs in the initial two cases, revealed the inferior aspects of the left inferior PVs had been partially covered using the ElePulse Catheter. This positioning was rectified, and PVI durability at 3 months increased to 100% (both per PV and patient) in the final 6 consecutive patients with 100% freedom from AF at 6 and 12 months. This was dubbed the “optimized subset” (Table 2).

There were also 7 (19.4%) zero-fluoroscopy patients (two in cohort 1, one in cohort 2 and four in cohort 3). Trans-esophageal echo and mapping were used to position the ElePulse catheter. At 3 months, in the zero-fluoroscopy patients there was a PVI durability of 96% per vein and 86% of the zero-fluoroscopy patients having all PVs isolated. All 7 patients had no AF recurrence at 6 and 12 months.

### Catheter positioning, learning curve and left atrial EAM

**Fig. 3:**
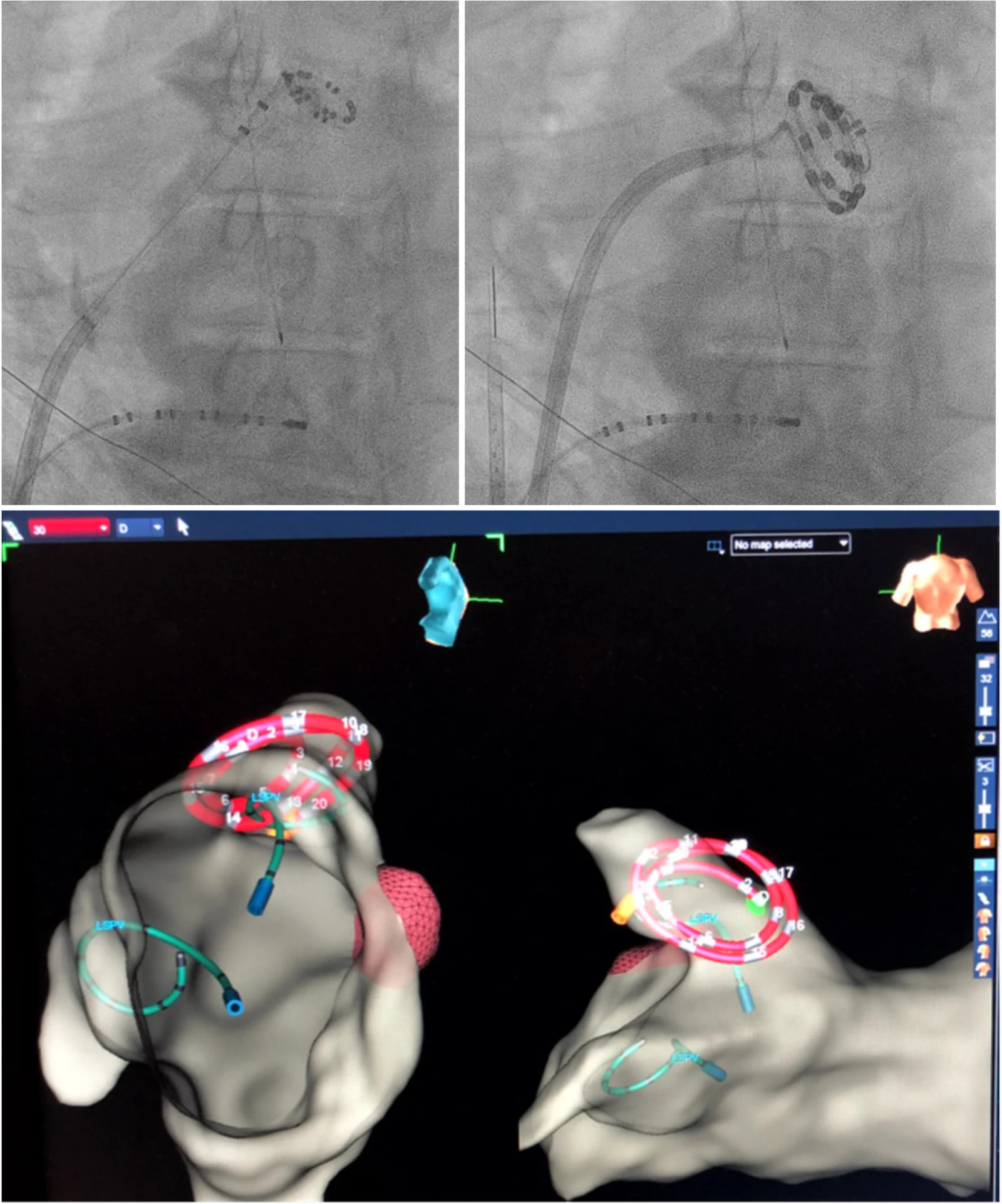
Appearance of PFA catheter positioned inside the left superior pulmonary vein. 3D (electroanatomic mapping) EAM and fluoroscopy were used to capture the catheter’s location. Alt text: Two x-ray images, one of the mapping catheter and one of the ablation catheter in the exact same position in the LUPV. The third figure is the Ensite map of the ablation catheter position

**Fig. 4:**
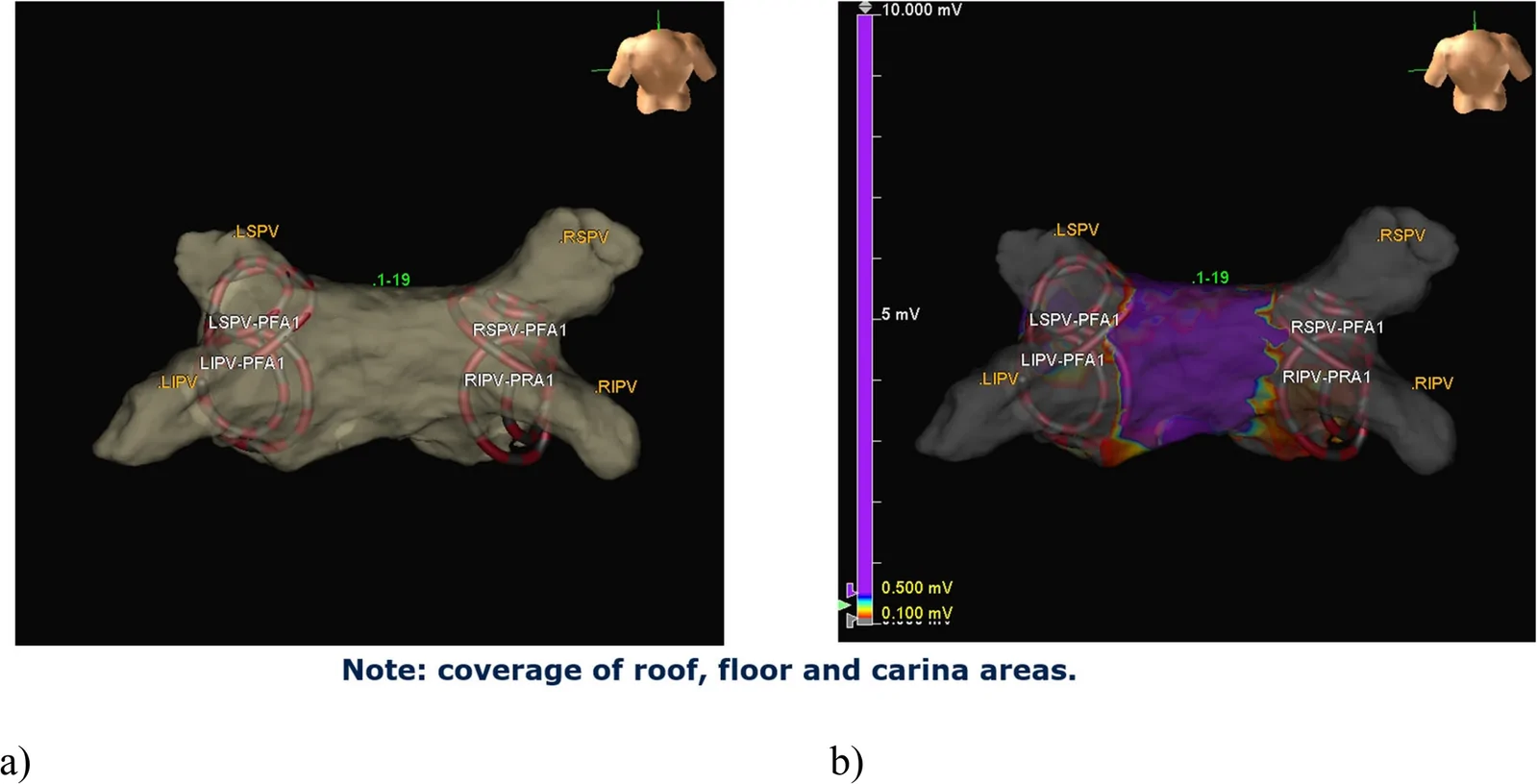
Optimal catheter positioning (shadowed) to achieve PVI. Typical (**a**) pre- and (**b**) post-ablation EAMs were performed to demonstrate the optimal catheter positions. Alt text: Figures demonstrate maps of left atria posteriorly with ideal positions for the ablation catheter and the ablated area in grey in the second figure

A comparison of ElePulse catheter positioning in left superior PV with the circular mapping catheter and the subsequent EAM in the left atrium is demonstrated in Fig. 3. Demonstration and explanation of contact parameters utilized in study are shown in Supplemental Fig. 2, with the impedance bar graphs for ablations that resulted in durable (Supplemental Fig. 2a) versus non-durable PVI (Supplemental Fig. 2b). Typical pre- and post-ablation maps along with typical catheter positions are also presented in Fig. 4. The learning curve for use of the ElePulse PFA catheter was rapid for the four operators in this study. All operators were experienced with radiofrequency ablation and the use of circular mapping catheters (> 250 cases) and 3 operators had > 100 cases of cryoablation experience.

The early 3-month re-mapping data from cohorts 1 and 2 pointed to the likelihood of PV reconnections at the carina, roof, and floor of PVs. Therefore, in cohort 3, particular attention was given to ablating these anatomical areas and was achieved by either overlapping catheter positions or by providing additional ablation treatments to confirm isolation of these areas. On average, there have been 2–4 additional shots given to properly cover the carinas and the roofs/floors, when necessary.

### Procedure times and fluoroscopy exposure

The results for procedure, ablation, mapping and left atrial dwell times and fluoroscopy exposure for each cohort and the overall study are presented in Table 3. For the overall study, the average procedural time was 128 ± 36 min, fluoroscopy exposure time, 23 ± 20 min, left atrial dwell time, 63 ± 31 min, mapping time, 34 ± 13 min and the overall PFA time, 6.1 ± 1.8 min. The overall number of treatments per PV was 2.1 ± 0.6. In addition, in the 7 zero-fluoroscopy cases performed the procedure time was 96 ± 20 min, including an ablation time of 5.2 ± 1.4 min and left atrial dwell time of 47 ± 19 min. Electrode collisions were detected in approximately 20% of applications. The generator was always able to flag the colliding electrodes so that the operator excluded them and continued with the treatment. When considering the procedure times, it must be acknowledged that this was a FIH study which aside from its learning curve, necessitated the creation of a high-density EAM, achieving optimal ElePulse catheter positioning and a protocoled, 20-min waiting period post-ablation.

### Primary safety endpoints

There were no detectable cases of PV stenosis (actively examined in all patients at index and at remap procedure times), transient ischemic attack or stroke, phrenic nerve/esophageal injury, cardiac perforation/tamponade or death. There was one serious adverse event in this study. An acute renal injury was detected on day 2 after catheter ablation. This resulted in a SAE rate of 2.8%, meeting the primary safety endpoint (SAE rate < 5%). The serious adverse event occurred in a 60-year-old male patient with PersAF. He had a baseline GFR of 36 mL/min/1.72 m^2^ and was not intravenously/orally hydrated peri-procedurally. This patient underwent two CT scans, both with contrast load, on the same day of the procedure. The first one failed to obtain images of adequate quality. He was in Cohort 1 “high-dose”, receiving only 6 PFA applications to achieve acute isolation of all four PVs and had durable PVI in all four PVs at his 3-month remapping study. He also remained free from AF at 6 and 12 months of follow-up. However, on post-procedure day 2, he presented with acute kidney injury with the serum creatinine increasing from 138 µmol/L (at baseline) to 720 µmol/L at its peak in the setting of acute oliguric renal failure. This condition eventually resolved with intravenous hydration and furosemide. There was no requirement for hemofiltration or dialysis A renal biopsy demonstrated red cell casts in the glomeruli and blood investigations demonstrated elevated hemolytic parameters. Given that the patient had pre-existing renal impairment, received two contrast-enhanced CT examinations on the day of the procedure, and did not receive periprocedural hydration, the event may be described as multifactorial acute kidney injury with a possible contribution from hemolysis.

There were no other adverse events encountered in this study.

### Primary efficacy endpoint

The primary efficacy endpoint of freedom from AF at 12 months was overall 92% (32/35 patients) as determined by clinical follow-up and monitoring, electrocardiograms and 24-h Holter monitoring. One patient was lost to follow-up at the 12-months but was free of AF at the 6-months. This achieved the study primacy efficacy endpoint of freedom from AF of > 50%.

### Secondary efficacy and outcome endpoints

The secondary efficacy endpoint of > 60% freedom from AF was achieved. The freedom from AF at 6 months was 94% (34/36 patients), as assessed by clinical, electrocardiographic and 24-h Holter monitoring. The overall PVI durability in all 36 patients was 82%/PV at 3-month re-mapping studies which achieved the predefined secondary endpoint of 3-month PVI durability/PV > 50%. The “medium-dose” cohort mitigated risks of renal injury with hydration and achieved favorable results as the “optimized subset”, given the 100% PVI durability and freedom from AF at 6 and 12 months.

### PFA-induced hemolysis

The results concerning hemolysis for cohort 2 and 3 are presented in Table 4. Although hemolysis was detected, it was subclinical, and the renal function actually improved post-procedure in the cohort 3 (“medium-dose”).

**Table 4:** Data for haemolytic parameters for cohort 2 and 3^a^

Item	Cohort 1	Cohort 2	Cohort 3
Hemoglobin relative change (%)	not measured	-13.2	-12.9
Total bilirubin relative change (%)	not measured	112	119
LDH pre-procedure (U/L)	not measured	196	209
LDH post-procedure (U/L)	not measured	343	385
LDH relative change (%)	not measured	75	92
eGFR pre-procedure (ml/min)	not measured	86.3	83.3
eGFR post-procedure (ml/min)	not measured	83.5	88.8
eGFR relative change (%)	not measured	-3.3	7.2
Creatinine relative change (%)	not measured	8.4	-9.2
BUN relative change (%)	not measured	22	20
Haptoglobin relative change (%)	not measured	-70.2	-41.9

^a^*BUN* blood urea nitrogen, *eGFR* estimated glomerular filtration rate, *LDH* lactate dehydrogenase

## Discussion

The FIH RESET-AF study demonstrated the ElePulse PFA system was safe and efficacious for the treatment of AF in patients with PAF and PersAF, achieving prespecified primary, secondary efficacy and safety endpoints. The current study demonstrated the feasibility of the studied device for pulmonary vein isolation associated with a high rate of durable isolation at 3 months. Despite being a FIH study, with iterative development of PFA dosing, high rates of freedom from AF were observed, particularly in the “optimized subset”, (Table 2). Only a single adverse event occurred during the study related to intravascular hemolysis, which was mitigated in subsequent patients using a strategy of pre-hydration and PFA dose regimen change.

The study protocol, endpoints, numbers enrolled, and outcomes were similar or superior to other FIH studies [9–11]. The RESET-AF study included a re-mapping procedure at 3 months which has been omitted in some FIH studies [12]. A 3-month mapping study is important to differentiate between reversible from irreversible electroporation effects, as durable PV isolation is emerging as an important predictor of long term outcomes [2]. Indeed, it was observed that regardless of the PFA dosing regimen, patients who had exhibited durable PVI at 3 months remained free from recurrent AF at 6 and 12 months. The 12-month freedom from recurrent AF in the current study was high compared to prior FIH PFA studies, 92% vs. 65–76% at 12 months [1, 2, 5]. Notwithstanding that 39% of the cohort consisted of patients with PersAF, often excluded in FIH studies. The higher rates of durable isolation and improved outcomes may be due to the one-shot circumferential device design and simultaneous rather than duty cycled PFA delivery producing a large contiguous electrical field. Additionally, the ElePulse PFA system has an impedance-based contact sensing, and routine use of LA pace capture and 3D mapping optimizing contact and catheter position likely contributed to the high rates of durable PV isolation observed.

Adverse outcomes encountered in other studies such as stroke and vascular site bleeding were not observed in the current study, possibly attributable to their use of larger bore transseptal introducers. In spite of geometrical similarities to some other circular multi-electrode PFA catheters, the ElePulse PFA system has a different waveform. Testing performed by independent researchers pointed out to significantly lower thermal effects with ElePulse PFA than with technologies currently on the market [13]. The ElePulse PFA catheter is compatible with a standard, commercially-available 8.5-Fr introducers which may decrease these complications. The learning curve was also rapid because the dual helical configuration of the ElePulse PFA catheter being similar to the familiar standard circular mapping catheter. In addition, the use of traditional parameters such as impedance for contact and nearfield bipolar pacing for real-time atrial signal evaluation, allow for immediate post-ablation assessment of the outcomes. A substantial proportion (19.4%) of the patients in this study underwent a zero-fluoroscopy ablation, guided by EAM and trans-esophageal imaging alone, which was facilitated by lack of need to visualise guide wires using this system. This demonstrates that the ElePulse PFA system can be adapted to a range of procedural workflows. Although the Ensite X EAM system (Abbott) was used exclusively in this study to localize the ElePulse catheter, following the current study, the Carto 3D EAM system (J&J MedTech) has also been successfully used with this catheter. The ElePulse catheter has also subsequently been used in one patient to perform posterior wall ablation using 4 to 6 additional applications (single ablations at each site for 15 s) achieving ablation of the entire posterior wall.

The only serious adverse event encountered was multifactorial acute kidney injury with a possible contribution from hemolysis. Based on this SAE, the PFA dose was adjusted and a periprocedural hydration protocol was implemented. At the time of this study, hemolysis was not an anticipated cause for renal impairment post-PFA. However, since the completion of this study, there have been an accumulation of human experiences with hemolysis [14]. While this is generally believed to be related to the extent of PFA, in the current study the adverse event occurred in a patient with only 6 applications. This may imply that the underlying renal impairment and IV contrast loading may have contributed to transient acute renal failure in our patient. After pre-hydration was implemented and PFA dosing was adjusted, no further clinically significant hemolysis was observed.

In summary, the CRC EP ElePulse catheter is a novel PFA system that uses a unique “single-shot” design to achieve PVI. Larger atria may require 1–2 additional applications to cover the carina. PV reconnection observed at 3 months were likely a consequence of reversible electroporation. As such, improvements in catheter positioning and relying on contact parameters helped to achieve 100% PVI durability at 3 months in the “optimized subset”.

### Limitations

The current study is limited by low numbers, short follow-up and no comparator group. However as a FIH proof-of-concept study, it is similar to previous FIH studies [9–11]. As this was also a proof-of-concept study, it was not powered to analyze outcome endpoints with statistical significance.

## Conclusion

The RESET-AF study is a proof-of-concept FIH study of the ElePulse PFA system in which 36 consecutive patients with symptomatic PAF and PersAF were treated, demonstrating the feasibility of this system for one-shot pulmonary vein isolation with high durability and low rates of AF recurrence. These findings suggest that this novel PFA system could be an effective and safe tool for catheter ablation treatment of atrial fibrillation.

### Clinical Perspectives

This FIH proof-of-concept study enrolled 36 patients for the ablative treatment of AF and demonstrated safety and efficacy using a novel single shot PFA catheter. The dosage and procedural characteristics were adjusted to establish an optimized cohort which achieved a very favorable 12-month outcome and PV durability at 3-months.

## Supplementary Information

Below is the link to the electronic supplementary material.

Supplementary Material 1

## Data Availability

Raw data cannot be shared openly in order to protect patients’ privacy. Also, some aspects of data and study are proprietary in confidence and cannot be publicly disclosed.
