# Supplementary material for: First-in-human experience from RESET-AF (Rapid onE-Shot Electroporation Trial for Atrial Fibrillation)
Source: J Interv Card Electrophysiol. 2026 Sep 17;69(6):1481–91. doi: 10.1007/s10840-026-02434-3 (PMC13646017; doi:10.1007/s10840-026-02434-3)
Supplement: Supplementary file 1 [file 10840_2026_2434_MOESM1_ESM.docx]

Supplemental Table 1. Study Endpoints^a^

Endpoints

Primary

Safety

ElePulse PFA SAEs at 7 days <5%

Procedure-related SAEs at 30 days <5%

Efficacy

Freedom from recurrent AF at 12 months of follow-up >50%

Secondary

Rate of pulmonary vein isolation durability at 3-month follow up >50%

Freedom from recurrent AF at 6 months of follow-up >60%

^a^ AF = atrial fibrillation; PFA = pulsed-field ablation; SAE = serious adverse event

Supplemental Table 2. Inclusion and Exclusion Criteria^a^

Criteria

Inclusion

Suitable candidate for intra-cardiac mapping and ablation for arrhythmias

History of recurrent symptomatic paroxysmal AF or persistent AF with ≥2 episodes reported within the 365 days (12 months) prior to enrollment

At least one episode of AF documented by Holter monitor, rhythm strip, trans-telephonic monitor (TTM), or 12-lead ECG prior to enrollment

Refractory to at least one Class I - IV anti-arrhythmic drug (AAD)

Eighteen (18) years of age or above

Competent and willing to provide written informed consent to participate in the study and agree to comply with the follow-up visits and evaluation

Exclusion

Previous left atrial ablation procedure

Intracardiac thrombus, tumor, or other abnormality that precludes catheter introduction and placement

Left atrial (LA) diameter > 50mm (if patients are symptomatic due to their AF burden and require an ablation for quality of life reasons, then a waiver to include larger LA diameters may be considered by the Sponsor)

Known severe cerebrovascular disease or history of cerebrovascular event (within 1 month)

Patients with severely impaired kidney function as measure by a Cockcroft-Gault Glomerular Filtration Rate (GFR) with a GFR ≤ 60 (previously ≤ 30 – see results section). This is calculated as follows for males:

GFR = (140 - age) x weight x .85 (for females)

PCr x 72

where age is specified in years, weight in kg, and PCr is Serum Creatinine in mg/dL. Female GFR is reduced by 15% of the above calculated value for males.

Serum creatinine greater than site’s normal range

Active gastrointestinal bleeding

Active infection or fever (>100.5°F/38°C)

Sepsis

Cardiac surgery within the past two months

Exposure to contrast medium ≥7 days prior to catheter ablation (this timeframe can be shorter if PI considers it safe to perform the ablation in <7 days)

Short life expectancy (<1 yr) due to other illnesses, such as cancer or pulmonary, hepatic, or renal disease

Significant anemia (hemoglobin <10.0 g/dL/100 g/L^b^)

Severe uncontrolled systemic hypertension (systolic pressure > 240 mmHg within the last 30 days

Documented anaphylaxis during previous exposure to angiographic contrast media (if contrast media not required patient meets eligibility)

Uncontrolled congestive heart failure (NYHA Class III or IV)

Unstable angina or acute myocardial infarction within the past three months

Bleeding, clotting disorders, or known thrombosis

Severe peripheral vascular disease

Uncontrolled diabetes

Rheumatic heart disease

Heart valve replacement

Mitral clip

Women who are of childbearing potential who are currently pregnant or not willing to use contraception for the duration of the study

Active participation in another investigational protocol currently or the last 30 days

Unable or unwilling to take anti-coagulants

Unwilling or unable to comply with any protocol or follow-up requirements

^a^ AF = atrial fibrillation; ECG = electrocardiogram; NYHA = New York Heart Association; PI = principal investigator

^b^ Value based on most recent result

Supplemental Table 3. Data Parameters Collected for Each Procedure^a^

Parameters

Catheter Identification

Model number

Lot/serial number

Procedural Data

PFA generator treatment recipe

Total number of PFA applications per vein

Overall total number of PFA applications

Total PFA time during the procedure

Mapping time

Fluoroscopy time

Procedure time (first catheter inserted to last catheter removed)

Impedance values during the procedure (Ω)

Activated Clotting Time levels and times taken

Ablation locations

Presence of char or coagulum formation and incidence(s) of steam pops

^a^ PFA = pulsed-field ablation

Supplemental Fig. 1. Relationship between PFA dose and hemolysis levels. An animal study was performed during the course of the first-in-human study to understand the relationship between the PFA dose and hemolysis levels (as described by (a) plasma-free hemoglobin [PFH] and (b) lactate dehydrogenase [LDH]). Swine #1 was ablated by delivering 8 “high-dose” PFA treatments without hydration. Swine #2 was ablated by delivering 16 “medium-dose” PFA treatments with 200 mL/h hydration. PFH and LDH were measured at baseline and after each treatment. Supplemental Figure 4 illustrates that despite the larger number of PFA treatments, swine #2 experienced lower PFH and LDH levels suggesting a likely safer strategy.

PFH: High vs Medium-dose treatments

PFH [mg/dL]

| PFA shot# | PFH_medium_dose |
| --- | --- |
| 0 | 3 |
| 1 | 78 |
| 2 | 111 |
| 3 | 195 |
| 4 | 66 |
| 5 | 102 |
| 6 | 210 |
| 7 | 237 |
| 8 | 72 |
| 9 | 84 |
| 10 | 84 |
| 11 | 120 |
| 12 | 126 |
| 13 | 174 |
| 14 | 186 |
| 15 | 204 |
| 16 | 222 |

| PFA shot# | PFH_high_dose |
| --- | --- |
| 0 | 2 |
| 1 | 54 |
| 2 | 54 |
| 3 | 96 |
| 4 | 144 |
| 5 | 168 |
| 6 | 234 |
| 7 | 336 |
| 8 | 366 |

LDH: High vs Medium-dose treatments

LDH [U/L]

| PFA shot # | LDH_medium_dose |
| --- | --- |
| 0 | 257 |
| 1 | 339 |
| 2 | 347 |
| 3 | 369 |
| 4 | 374 |
| 5 | 410 |
| 6 | 406 |
| 7 | 407 |
| 8 | 406 |
| 9 | 412 |
| 10 | 397 |
| 11 | 413 |
| 12 | 432 |
| 13 | 464 |
| 14 | 462 |
| 15 | 480 |
| 16 | 470 |

| PFA shot # | LDH_high_dose |
| --- | --- |
| 0 | 337 |
| 1 | 491 |
| 2 | 542 |
| 3 | 516 |
| 4 | 489 |
| 5 | 555 |
| 6 | 603 |
| 7 | 650 |
| 8 | 679 |

Supplemental Fig. 2. Impedance Contact Information for each PFA electrode. (a) illustrates an example of an impedance trend in a durable PVI; (b) exemplifies an impedance trend in a PV that had reconnected.

 (a)

 (b)
